# SGLT2 inhibitors: a novel therapy for cognitive impairment via multifaceted effects on the nervous system

**DOI:** 10.1186/s40035-024-00431-y

**Published:** 2024-08-09

**Authors:** Jiaqi Mei, Yi Li, Liyan Niu, Ruikai Liang, Mingyue Tang, Qi Cai, Jingdong Xu, Deju Zhang, Xiaoping Yin, Xiao Liu, Yunfeng Shen, Jianping Liu, Minxuan Xu, Panpan Xia, Jitao Ling, Yuting Wu, Jianqi Liang, Jing Zhang, Peng Yu

**Affiliations:** 1 Department of Endocrinology and Metabolism, The Second Affiliated Hospital of Nanchang University, Nanchang, China; 2 Huan Kui College of Nanchang University, Nanchang, China; 3 The Second Clinical Medical College of Nanchang University, The Second Affiliated Hospital of Nanchang University, Nanchang, China; 4 Queen Mary College of Nanchang University, Nanchang, China; 5 Food and Nutritional Sciences, School of Biological Sciences, The University of Hong Kong, Pokfulam Road, Hong Kong, China; 6 Department of Neurology, Affiliated Hospital of Jiujiang University, Jiujiang, China; 7 Department of Anesthesiology, The Second Affiliated Hospital of Nanchang University, Nanchang, China

**Keywords:** Cognitive impairment, Diabetes, Sodium-glucose cotransporter-2, SGLT2 inhibitor, Neuron

## Abstract

The rising prevalence of diabetes mellitus has casted a spotlight on one of its significant sequelae: cognitive impairment. Sodium-glucose cotransporter-2 (SGLT2) inhibitors, originally developed for diabetes management, are increasingly studied for their cognitive benefits. These benefits may include reduction of oxidative stress and neuroinflammation, decrease of amyloid burdens, enhancement of neuronal plasticity, and improved cerebral glucose utilization. The multifaceted effects and the relatively favorable side-effect profile of SGLT2 inhibitors render them a promising therapeutic candidate for cognitive disorders. Nonetheless, the application of SGLT2 inhibitors for cognitive impairment is not without its limitations, necessitating more comprehensive research to fully determine their therapeutic potential for cognitive treatment. In this review, we discuss the role of SGLT2 in neural function, elucidate the diabetes-cognition nexus, and synthesize current knowledge on the cognitive effects of SGLT2 inhibitors based on animal studies and clinical evidence. Research gaps are proposed to spur further investigation.

## Introduction

Sodium-glucose cotransporter-2 (SGLT2) inhibitors are a category of antidiabetic drugs that act on the SGLT2 proteins to reduce glucose reabsorption and lower plasma glucose concentrations [1]. The global prevalence of diabetes is on the rise. By 2040, about 642 million people are projected to be affected by diabetes mellitus. According to a large U.S. veterans registry, the prevalence of concurrent dementia and cognitive impairment among individuals with diabetes was 13.1% in the 65–74 age group and 24.2% in those aged 75 years and older [2]. Cognitive impairment is a complication of diabetes mellitus, with neurodegenerative alterations being associated with the pathogenic mechanisms of cognitive impairment. Consequently, there has been an increasing focus on the impact of antidiabetic drugs on cognitive dysfunction [3]. Neurodegenerative changes are associated with the pathogenesis of cognitive impairment, whereas SGLT2 inhibitors can protect the nervous system through anti-inflammatory and anti-oxidative stress effects [4, 5]. Accumulating evidence from both clinical and experimental studies has shown that SGLT2 inhibitors have a therapeutic potential for cognitive impairment [6–14]. Current drugs for cognitive impairment mainly act by inhibiting cholinesterase activity and glutamate excitotoxicity, and most of the drugs such as donepezil and lisdexamfetamine have side effects such as decreased hepatic function and gastrointestinal discomfort [15, 16]. SGLT2 inhibitors expand the therapeutic approaches for cognitive disorders by reducing neuroinflammation, enhancing cerebral glucose metabolism and energy availability, limiting amyloid production and aggregation, and modulating neurotrophic factor production to support neural regeneration [17]. In this article, we elucidate the pathophysiological role of SGLT2 in the nervous system, discuss the association between diabetes mellitus and diverse neurodegenerative diseases, and analyze current clinical investigations into SGLT2 inhibitors for neurodegenerative disorder management. The mechanisms by which these inhibitors promote neuronal survival are also discussed.

## Pathophysiological role of SGLT2 in the nervous system

### Physiological roles of SGLT2

SGLT2 is a glucose transporter primarily expressed in the proximal tubules of the kidney [18]. SGLT2 is also prevalent throughout the central nervous system (CNS), including the brain parenchyma and blood–brain barrier (BBB) [19–22]. In the BBB, SGLT2 expression in endothelial cells facilitates selective glucose transport, which is crucial for maintaining cerebral glucose levels [23].

SGLT2 expression in the brain parenchyma is predominantly observed in the hippocampus and cerebellum, where it is implicated in learning, appetite regulation, energy balance, and central cardiovascular and autonomic control [3]. Its presence in the hippocampus has also been linked to synaptic plasticity. Studies have demonstrated that SGLT2 inhibitors enhance insulin sensitivity in the brains of obese rats and improve brain function by mitigating inflammation, apoptosis, and oxidative stress, which markedly boost hippocampal synaptic plasticity [24].

SGLT2 is mainly expressed in pericytes, facilitating glucose transport to these cells for their nourishment and metabolic functions. At this point, the pericytes share the glucose they have taken up with the nearby astrocytes in addition to using it for their own growth [25]. SGLT2 is also central to neurons, where it is present in cell bodies, axons, and dendrites. There is a hypothesis that SGLT2 inhibitors contribute to glucose absorption in both glial cells and neurons, suggesting a potential therapeutic mechanism. SGLT2 plays a crucial role in neuronal survival, particularly noted with increased expression under stroke or ischemic conditions [26]. Recent research revealed that treatment with the SGLT2 inhibitor dapagliflozin in dietary-induced diabetic mice, leads to elevated expression of doublecortin and synaptophysin, which are markers indicative of neurogenesis or synaptogenesis [27].

### Pathological roles of SGLT2

**Fig. 1:**
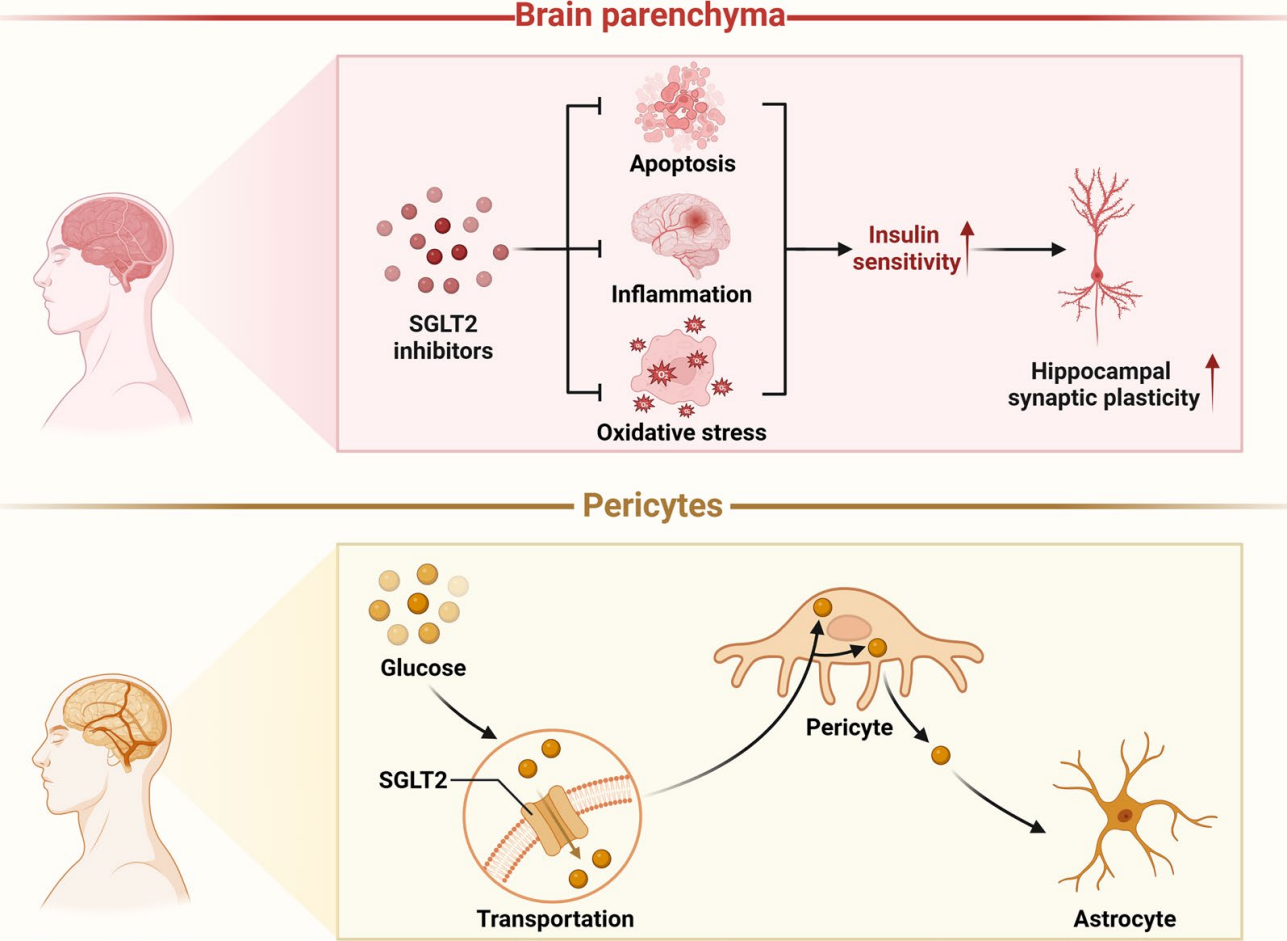
Physiological roles of SGLT2 in the brain. SGLT2 is mainly expressed in pericytes and brain parenchyma. SGLT2 expressed in pericytes facilitates glucose transport to support their nourishment and metabolic functions, with the additional role of distributing glucose to adjacent astrocytes. SGLT2 inhibitors enhance insulin sensitivity in the brains of obese rats by mitigating inflammation, apoptosis, and oxidative stress, markedly improving hippocampal synaptic plasticity

Associations between SGLT2 expression and various pathologies have been substantiated in multiple studies. Post-mortem analyses of human brain tissues have revealed a marked elevation of SGLT2 level in areas of cerebral damage [28]. Umino et al. documented that high-glucose conditions lead to increased SGLT2 expression and increased renal threshold for glucose, resulting in enhanced glucose reabsorption, exacerbating hyperglycemia [29, 30]. Additionally, research has demonstrated a significant surge in sodium ion influx into neuronal cells mediated by SGLT2 during cerebral ischemia, which amplifies neuronal damage [31]. Moreover, D'Onofrio et al. noted a pronounced increase in SGLT2 expression within atherosclerotic plaques of diabetic patients compared to non-diabetics [32]. These findings imply that pathogenic factors, including brain injury and hyperglycemia, would stimulate SGLT2 expression, further leading to a cascade of detrimental effects such as enhanced glucose reabsorption, neuronal damage, and atherogenesis (Fig. 1).

## Relationship between diabetes and multiple neurodegenerative diseases and SGLT2 intervention studies

### Effects of diabetes on the nervous system

Diabetic neuropathy represents a significant complication of diabetes mellitus, with peripheral neuropathy being the most frequent presentation. Hyperglycemia is identified as the primary cause of this condition [33, 34]. The disease is characterized by marked axonal degeneration and segmental demyelination involving peripheral sensory and motor nerves. Damage to motor nerves may lead to neuromuscular atrophy with concomitant decreases in muscular endurance, size, and metabolism, so the main symptoms of the disease are muscle atrophy and sensory abnormalities [35, 36]. Diabetes can lead to neurological damage through multiple pathological mechanisms, including oxidative stress and neuroinflammation. Strom et al. recently reported that reduced levels of extracellular superoxide dismutase (SOD) and glutathione (GSH) are significantly correlated with diabetic neuropathy in patients with diabetes [37]. However, there is also a study showing a significant increase of SOD and a significant decrease of GSH levels in erythrocytes from diabetic patients. Such partially contradictory experimental results suggest that the status of oxidative stress varies among diabetic patients. More studies are needed to confirm this observation [38]. These findings support the hypothesis that oxidative stress is a critical contributor to diabetic peripheral neuropathy. Furthermore, elevated blood levels of glucose may lead to activation of the cyclooxygenase-2 pathway within the microvasculature, which in turn promotes inflammation and oxidative stress in peripheral nerves[39].

In addition to the role in neurological impairment, accumulating evidence suggests that hyperglycemia is involved in BBB damage [40]. Hyperglycemia promotes production of reactive oxygen species (ROS), which leads to activation of nuclear factor κ-light chain enhancer of activated B cells (NF-κB), activation protein-1, and the signal transducer and activator of transcription (STAT) pathways, resulting in upregulation of inflammatory cytokines. During the inflammatory response, disruption of components of the BBB such as astrocytes and basement membranes, and downregulation of expression of some tight junction proteins such as claudin-5 and occludins, promote leukocyte extravasation, increase the diffusion of solutes across the BBB, and permit the entry of pathogens and toxins into the CNS [41, 42].

**Fig. 2:**
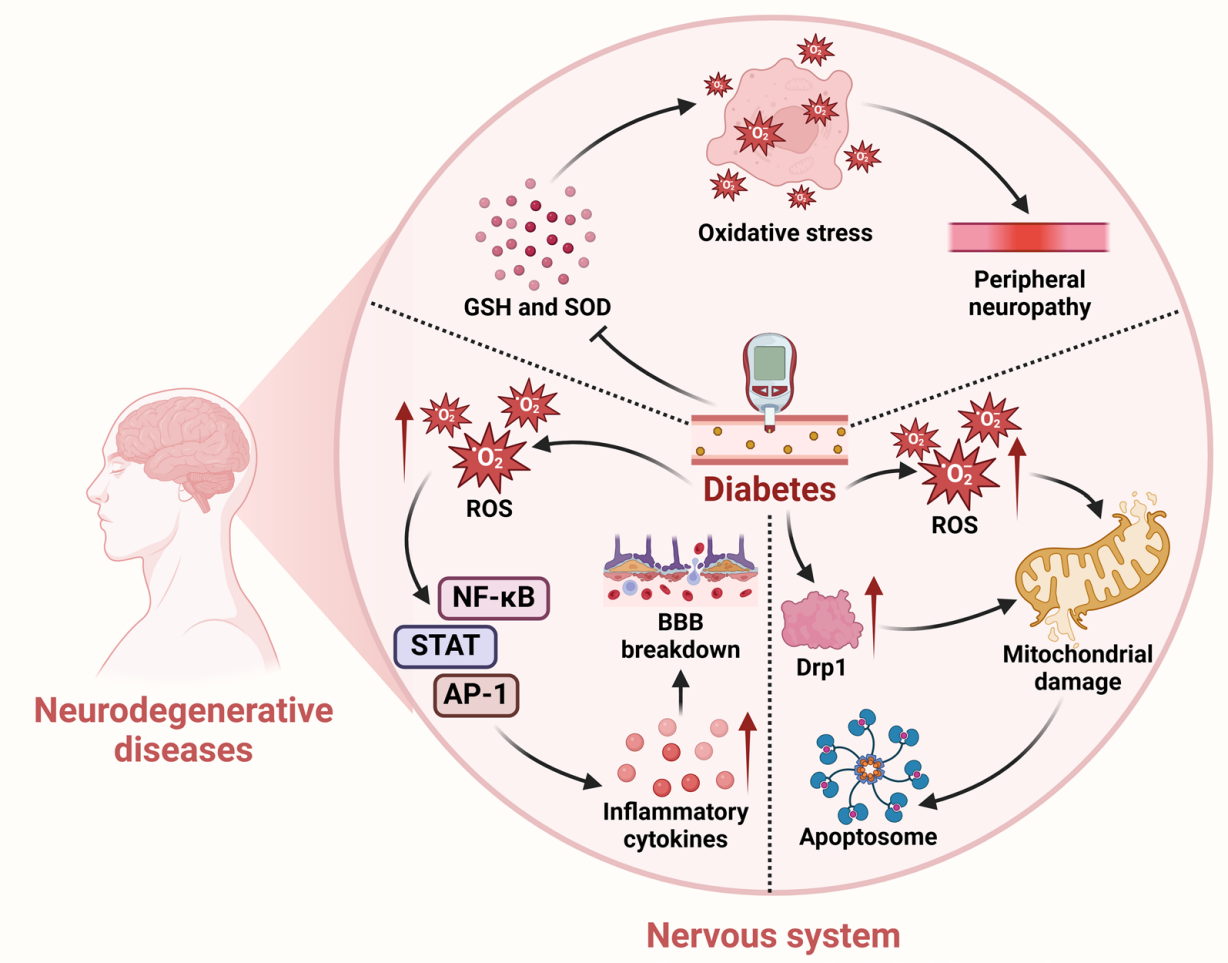
Diabetes mellitus affects the nervous system. Diabetes can affect the nervous system through three mechanisms. First, it reduces the levels of extracellular superoxide dismutase (SOD) and glutathione (GSH), which in turn promotes inflammation and oxidative stress in peripheral nerves. Second, it promotes the production of reactive oxygen species (ROS), leading to the activation of nuclear factor κ-light chain enhancer (NF-κB), activation protein-1 (AP-1), and the signal transducer and activator of transcription (STAT) pathways, resulting in increased inflammatory cytokines and then increased BBB breakdown. Third, it increases the level of dynamin-related protein 1 (Drp1) in mitochondria within neurons and stimulates the overproduction of ROS, resulting in impaired mitochondrial morphology and function, which in turn leads to neuron apoptosis

Hyperglycemia is also correlated with neuronal abnormality. Studies have shown that hyperglycemia increases levels of dynamin-related protein 1 in mitochondria of neurons, resulting in impaired morphology and function of mitochondria, which in turn leads to synaptic dysfunction [43]. Moreover, hyperglycemia accelerates neuronal apoptosis through overproduction of ROS and free radicals. The resulting oxidative stress activates effector proteins, which compromise the mitochondrial membrane potential, causing mitochondrial swelling and increased permeability. As a result, the caspase-3 pathway is activated, which promotes the release of apoptogenic proteins like cytochrome *c* from the mitochondria to the cytosol [44, 45]. Hyperglycemia further promotes apoptosis by boosting caspase activity, leading to cellular events such as DNA fragmentation, degradation of structural and nuclear proteins, protein cross-linking, apoptotic body formation, and ultimately phagocytic absorption (Fig. 2) [45–47].

### The relationship between diabetes and cognitive decline

Diabetes significantly elevates the risk of Alzheimer's disease (AD), with insulin resistance or type 2 diabetes mellitus (T2DM) present in approximately 80% of AD patients [48, 49]. The duration and management of diabetes, along with glycemic variability, correlate with cognitive functions [50–52]. Diabetes contributes to the development of mild cognitive impairment, characterized by reduced processing speed, and impaired memory, attention, and executive abilities [53, 54]. Several mechanisms by which diabetes impairs cognition have been identified.

#### Diabetes triggers neuroinflammation

Diabetes can induce neuroinflammation by activating the NF-κB pathway, increasing production of proinflammatory cytokines and toll-like receptor expression, and improving oxidative stress and inflammasome activation. Neuroinflammation is pivotal in AD pathogenesis and involves the activation of brain-resident inflammatory cells like microglia and astrocytes. These cells exacerbate the inflammatory state by releasing cytokines, interleukins and chemokines, which may surround and amplify neurofibrillary tangles and senile plaques characteristic of AD [55, 56].

#### Diabetes accelerates vascular aging

Diabetes can accelerate vascular aging through inflammatory responses that lead to elevated production of cytokines such as tumor necrosis factor-α (TNF-α) and interleukin (IL)-6. The resulting vascular changes can diminish cerebral blood flow and perfusion, leading to ischemic damage, white matter changes, basal ganglia diffuses and neuronal loss and apoptosis, impairing executive cognitive functions [57–59].

#### Diabetes leads to hyperglycemia

Hyperglycemia due to diabetes leads to neuronal glucotoxicity, which is referred to as the detrimental effect of excessive glucose [60]. Glucotoxicity leads to abnormal intracellular accumulation of methylglyoxal, which impairs dopaminergic neuron survival [61]. The loss of dopaminergic neurons then leads to memory and reward dysfunction [62]. Methylglyoxal also promotes endogenous non-enzymatic glucose oxidation of proteins, lipids, and nucleic acids with consequent formation of advanced glycosylation end-products (AEGs). Elevated plasma levels of AEGs may be associated with atrophy of the grey matter, and accumulation of AEGs may lead to neuronal dysfunction and increased vulnerability [63, 64]. The accumulation of AEGs further activates the receptor for advanced glycosylation end products (RAGE), which promotes the activation of ROS, leading to neuronal death [65]. RAGE also binds to amyloid β (Aβ) and exacerbates its aggregation, resulting in hyperphosphorylation of tau and formation of senile plaques, ultimately leading to cognitive impairment [66].

#### Diabetes promotes oxidative stress

Activation of the pro-oxidative protein kinase C pathway, the hexosamine pathway, and the polyol metabolic pathway in the development of diabetes contributes to oxidative stress [67], promoting the production of NF-κB and NADPH oxidase 2 (NOX2). NF-κB is a key promoter of oxidative stress and neuronal death following Aβ peptide stimulation of microglia and astrocytes in AD. Unwanted NF-κB transcriptional activation can also trigger a variety of modifications in the expression of genes implicated in the cognitive deterioration associated with diabetes [68]. NOX2 can contribute to oxidative stress by promoting ROS production and thus oxidative stress. In addition, enhanced NOX2 expression in vascular endothelial cells may lead to increased cerebral vascular permeability, promoting leukocyte adhesion and CNS inflammation [69]. There is a close relationship between Aβ level and NOX2 activity, and NOX2-derived oxidative stress plays a major role in mediating Aβ-induced neuronal death and neurovascular dysfunction [70, 71]. Nox2 deletion in Tg2576 AD mice rescues the cerebrovascular dysfunction and behavioral deficits. In addition, a peptide inhibitor of NADPH oxidase also ameliorates neurovascular dysfunction. It inhibits the overproduction of NOX-derived ROS, thereby reducing amyloid precursor protein (APP) expression and Aβ-induced neurovascular dysfunction [72].

#### Treatment of diabetes triggers hypoglycemia

Hypoglycemia, a common complication during treatment of diabetes, is also one of the risk factors for cognitive dysfunction. Studies have shown that 58%–64% of patients receiving insulin and non-insulin treatments require medical assistance to treat hypoglycemia over a period of 6–12 months [73]. Hypoglycemia may lead to neuronal death by promoting oxidative stress, zinc release, poly(ADP-ribose) polymerase 1 activation, and mitochondrial dysfunction, and the neuronal damage and cognitive decline caused by severe hypoglycemia are exacerbated by diabetes [74]. Severe hypoglycemia causes damage specifically in the hippocampus involved in memory and learning, leading to impaired cognitive function [73].

#### Insulin resistance often accompanies diabetes

Insulin resistance is one of the earliest defects in the pathogenesis of T2DM and one of the features of AD [68, 75]. Chronic peripheral hyperinsulinemia due to insulin resistance ultimately reduces brain insulin levels and leads to desensitization of neuronal insulin receptors. This may result in reduced clearance of Aβ peptides and increased hyperphosphorylation of τ proteins, leading to the formation of neurofibrillary tangles, in turn contributing to cognitive impairment [76, 77].

#### Diabetic patients often show dysfunctional lipid metabolism

**Fig. 3:**
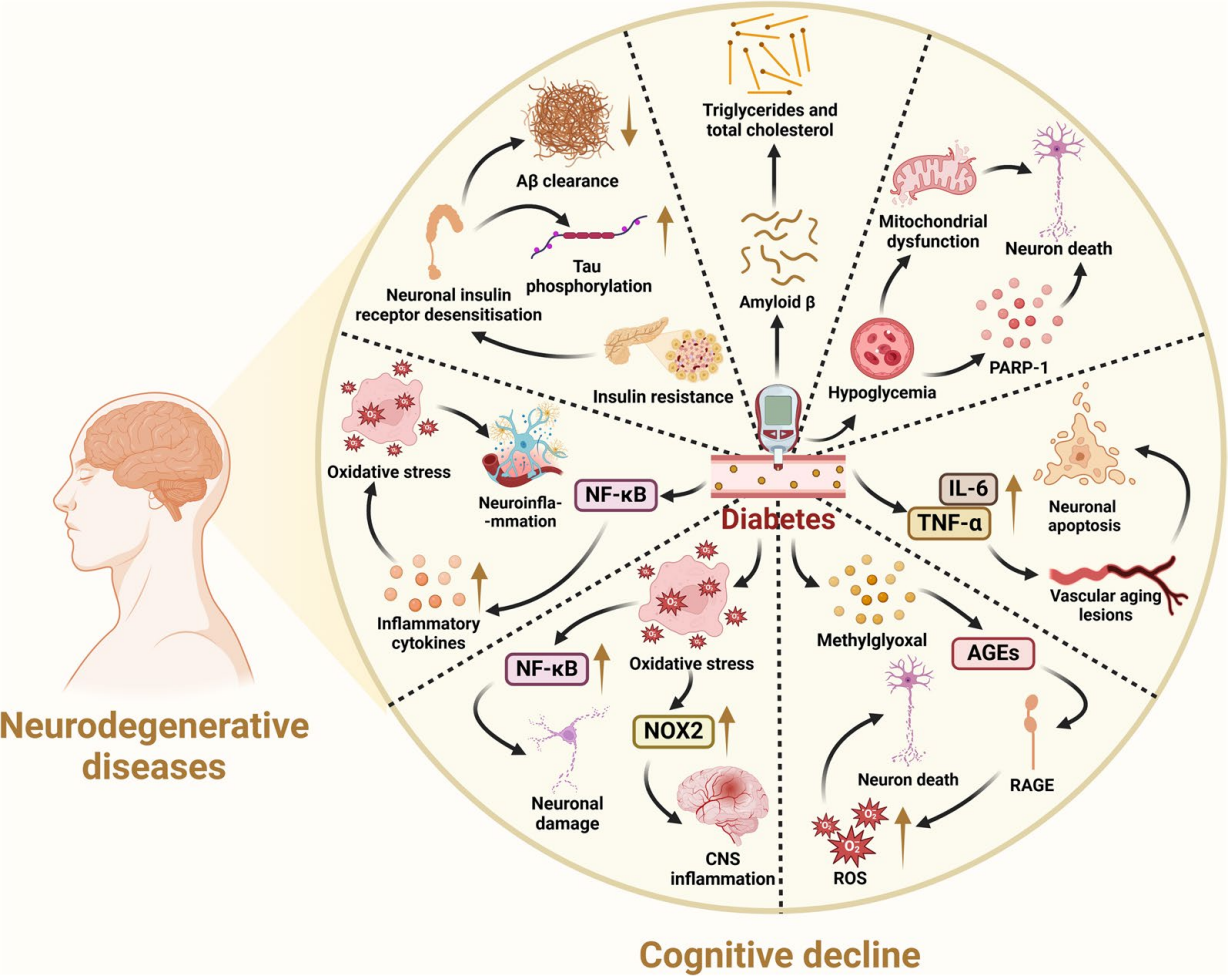
Diabetes mellitus is associated with cognitive decline. Diabetes can affect cognitive decline through the following mechanisms. First, it induces neuroinflammation by activating the NF-κB pathway, enhancing proinflammatory cytokine production, and stimulating oxidative stress and inflammasome activation. Second, it accelerates vascular aging through inflammatory responses that elevate cytokines such as tumor necrosis factor-α (TNF-α) and interleukin-6 (IL-6), resulting in neuronal loss and apoptosis. Third, it leads to neuronal glucotoxicity, which leads to an abnormal intracellular accumulation of methylglyoxal. Methylglyoxal promotes the formation of advanced glycosylation end-products (AGEs) and activates the receptor for advanced glycosylation end products (RAGE), leading to activation of ROS and thus neuronal death. Fourth, it promotes oxidative stress and facilitates the production of NF-kB and NADPH oxidase 2 (NOX2), which lead to neuronal damage and neuroinflammation. Fifth, treatment of diabetes may trigger hypoglycemia. Hypoglycemia promotes neuronal death by inducing activation of poly(ADP-ribose) polymerase 1 (PARP-1) and mitochondrial dysfunction. Sixth, insulin resistance ultimately reduces brain insulin levels and leads to neuronal insulin receptor desensitization, resulting in reduced clearance of Aβ peptides and increased hyperphosphorylation of tau protein. Last, triglyceride (TG) and cholesterol levels are increased under the condition of diabetes, which facilitate Aβ overproduction and deposition

Diabetes frequently co-occurs with lipid metabolism disorders, characterized by increased levels of triglyceride (TG) and cholesterol. These alterations can disrupt APP metabolism, facilitate overproduction and deposition of Aβ peptides, and are significantly associated with cognitive deficits in diabetic patients [58]. Concurrently, elevated TG levels may compromise *N*-methyl-*D*-aspartate receptor-dependent synaptic potentiation in the hippocampus, further contributing to cognitive decline (Fig. 3) [78].

## Mechanisms involved in the regulation of neuronal survival by SGLT2 inhibitors

### SGLT2 inhibitors suppress neuroinflammation

Neuroinflammation is closely related to cognitive deficits [5]. Suridjan et al. observed a positive correlation between neuroinflammation and the severity of cognitive impairment when comparing cognitively normal controls and individuals with AD [79]. Notably, recent investigations have highlighted the anti-inflammatory effects of SGLT2 inhibitors [80].

NOD-like receptor thermal protein domain associated protein 3 (NLRP3) inflammasomes are pivotal in the innate immune system, and their dysregulation contributes to AD [81]. Tejera et al. found that the NLRP3 inflammasomes mediate the systemic inflammation (lipopolysaccharide)-induced reduction of microglial clearance of Aβ in APP/PS1 mice [82]. A comparative study by Kim et al. treated patients with T2DM and high cardiovascular risk with either SGLT2 inhibitors or sulfonylureas for 30 days. Results showed that in addition to glucose-lowering capacity, SGLT2 inhibitors markedly suppressed NLRP3 inflammasome activation in macrophages. This suppression was mediated by elevations in serum β-hydroxybutyrate and reductions in serum insulin, which in turn inhibited the NLRP3/IL-1/TNF-α/miR-501-3p/ZO-1 axis and associated neuroinflammation [83]. Furthermore, SGLT2 inhibitors attenuate inflammation and neuronal damage through inhibition of ROS-dependent neuronal apoptosis, downregulation of the phosphatidylinositol 3-kinase (PI3K)/Akt/GSK-3β signaling pathway, and inhibition of the NF-κB pathway and TNF-α activation [84].

### SGLT2 inhibitors improve cerebral glucose metabolism and energy supply

SGLT2 inhibitors selectively target SGLT2 on the luminal surface of proximal renal tubules, inhibiting glucose reabsorption and promoting urinary glucose excretion. Consequently, this decreases plasma levels of glucose and glycated hemoglobin in individuals with T2DM [85, 86], which can lead to a decrease of insulin levels and an increase of glucagon release, promoting production of ketone body [87]. SGLT2 inhibitors also inhibit SGLT2-mediated reabsorption of sodium ions, leading to increased sodium ion levels in renal tubular fluid, which in turn increases the electrochemical gradient driving carrier-mediated reabsorption of negatively charged ketones [88]. As a result, brain metabolism shifts from utilizing carbohydrates to utilizing ketones [89]. Additionally, SGLT2 inhibitors potentially enhance β-oxidation in the hepatic tissue, which encourages the production of ketone bodies [90].

In most neurodegenerative diseases, glucose uptake and transport is impaired, resulting in an energy deficit state within the brain that exacerbates disease progression [91]. Interestingly, while glucose metabolism is impaired, ketone body metabolism typically remains unchanged, providing an alternative energy source for the brain [92]. Ketone bodies not only serve as alternative fuels for neuronal activities but also enhance mitochondrial function and efficiency [91]. Compared to glucose, ketone bodies are used more efficiently by neurons, astrocytes and oligodendrocytes, offering a more efficient energy provision for brain cells [93]. Consequently, SGLT2 inhibitors can support neuronal survival by optimizing cerebral glucose metabolism and supplementing energy supplies.

### SGLT2 inhibitors modulate neurotrophic factors and synaptic plasticity

The SGLT2 inhibitors can increase neurotrophic factors such as brain-derived neurotrophic factor (BDNF), nerve growth factor (NGF), and glial cell line-derived neurotrophic factor (GDNF) by alleviating neuronal ROS/oxidative stress and subsequently restoring the DJ-1/Nrf2 pathway [84, 94, 95]. BDNF critically governs the development of brain circuits, synaptic plasticity, neuroregeneration, and neuroprotection, playing a pivotal role in synaptic transmission [96, 97]. NGF is instrumental in the axonal branching of developing neurons in the peripheral nervous system, contributing significantly to synaptic plasticity [98].

In a previous study, dapagliflozin administration in diabetic mice induced notable upregulation of both doublecortin (number of immature neurons) and synaptophysinn (synaptic density), indicating potential drug efficacy in facilitating neurogenesis and the rehabilitation of synaptic density [27]. But this study did not explore in detail the specific mechanism underlying the enhancement of doublecortin and synaptophysin expression. In addition, SGLT2 inhibitors enhance insulin sensitivity in the brains of obese mice by reducing neuroinflammation, apoptosis, and oxidative stress, which in turn enhances mitochondrial brain function and significantly increases hippocampal synaptic plasticity [99]. These findings underscore the potential of SGLT2 inhibitors to modulate neuronal survival through regulation of neurotrophic factors and enhancement of synaptic plasticity.

### SGLT2 inhibitors suppress the production and aggregation of amyloid

Aβ deposition is a hallmark and key event of AD [100]. SGLT2 inhibitors appear to mitigate Aβ production by activating the adenosine monophosphate-activated protein kinase (AMPK) signaling pathway. SGLT2 inhibitors stimulate AMPK via hepatic kinase B1 [101], which reduces amyloid formation in neurons through a variety of ways.

Aβ is produced from sequential cleavage of APP by β and γ secretases [102]. Beyond reducing β-secretase-mediated cleavage of APP in neurons, AMPK also modulates the expression levels of α and β secretases, thereby influencing APP processing. Moreover, AMPK activation decreases the level of sphingomyelin in neuronal lipid rafts, consequently affecting the amyloidogenic processing of APP in lipid rafts [103].

Vascular dysfunction may also be associated with amyloid deposition [104]. Studies have shown that cerebrovascular diseases such as chronic cerebral underperfusion and BBB deterioration, together with cardiovascular diseases such as atrial fibrillation and heart failure, and other vascular risk factors, may promote the deposition of Aβ [105–107]. SGLT2 inhibitors can effectively reduce these risk factors by lowering blood pressure, inhibiting adipokines and cytokine-mediated inflammation, and reducing ROS production and NLRP3 inflammasome activity [108]. However, currently there is no direct evidence that SGLT2 inhibitors inhibit Aβ production and aggregation by improving vascular factors. More studies are needed to explore the involvement of this pathway. Meanwhile, overactivation of the mammalian target of rapamycin (mTOR) leads to rapid loss of brain function in rats, promoting Aβ deposition, BBB permeability, and tau protein hyperphosphorylation. In contrast, SGLT2 inhibitors can decrease mTOR activity to a normal level, halting the onset or progression of AD [99]. Given that Aβ contributes to neuronal apoptosis [109], the inhibition of amyloid production by SGLT2 inhibitors suggests a protective role in neuronal survival.

### Neuroregenerative effects of SGLT2 inhibitors

A previous animal study shows that the inhibition of C-reactive protein by SGLT2 inhibitor empagliflozin promotes macrophage polarization to the M2 phenotype [110]. The M2 macrophages play a pivotal role in nerve regeneration. They synthesize hypoxia-inducible factor 1 subunit-α in response to injury-induced hypoxia, subsequently leading to elevated expression of vascular endothelial growth factor A, a key regulator of endothelial cell proliferation and migration [111]. Meanwhile, empagliflozin significantly increases tissue levels of BDNF, NGF, and GNDF in diabetic mice [84, 94, 95], all neurotrophic factors playing important roles in nerve regeneration [112]. For example, BDNF can stimulate the intrinsic regenerative capacity of neurons by promoting mRNA expression that provokes the regeneration capacity of neurons [113]. NGF can bind pro-myosin receptor kinase A, activating a cascade of molecular pathways such as phospholipase C-γ, MAPK (mitogen-activated protein kinase)/Erk, and PI3K pathways. These pathways collectively contribute to the induction of neural regeneration [114]. However, direct evidence showing that SGLT2 inhibitors promote neural regeneration by modulating M2 polarisation in macrophages or the levels of BDNF, NFG, and GNDF proteins is lacking.

### Clinical trials on SGLT2 inhibitors

**Fig. 4:**
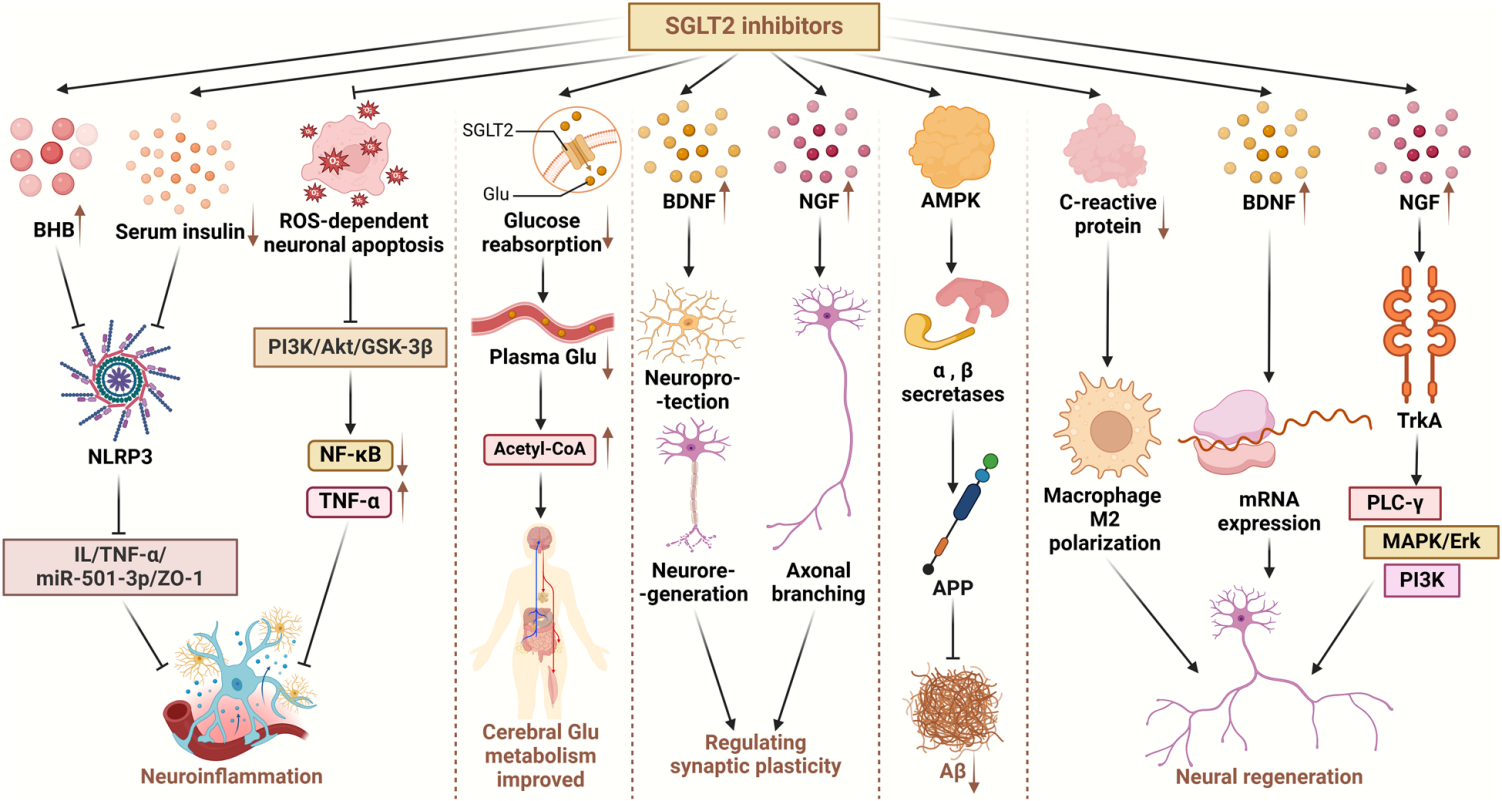
Mechanisms underlying the regulation of neuronal survival by SGLT2 inhibitors. (1) SGLT2 inhibitors suppress NLRP3 inflammasome activation in macrophages by elevating serum β-hydroxybutyrate (BHB) and reducing serum insulin, which in turn inhibit the NLRP3/IL-1/TNF-α/miR-501-3p/ZO-1 axis. Furthermore, SGLT2 inhibitors inhibit the ROS-dependent neuronal apoptosis, downregulate the PI3K/Akt/GSK-3β signaling pathway, and inhibit the NF-κB pathway and TNF-α activation. These pathways inhibit the neuroinflammation. (2) SGLT2 inhibitors target SGLT2 on the proximal renal tubules, reduce glucose reabsorption and promote urinary glucose excretion, which lead to the shift of brain metabolism from utilizing carbohydrates to fatty acid oxidation, and thus optimizes cerebral glucose metabolism. (3) SGLT2 inhibitors increase levels of neurotrophic factors like brain-derived neurotrophic factor (BDNF) and nerve growth factor (NGF), contributing to synaptic plasticity together. (4) SGLT2 inhibitors activate adenosine monophosphate-activated protein kinase (AMPK) via liver kinase B1, and AMPK modulates the expression of α and β secretases, thereby reducing Aβ generation from amyloid precursor protein (APP). (5) SGLT2 inhibitors promote macrophage polarization to the M2 phenotype, and M2 macrophages promote nerve regeneration. Meanwhile, SGLT2 inhibitors significantly increase tissue BDNF and NGF levels. BDNF can promote mRNA expression that provokes intrinsic regeneration capacity of neurons and NGF can bind to pro-myosin receptor kinase A (TrkA) to activate a cascade of molecular pathways to induce neural regeneration

The research into the numerous therapeutic benefits of the SGLT2 inhibitors outlined above for neurodegenerative illnesses is no longer in the experimental stage, and data from a variety of clinical trials suggest that they have the potential to treat these diseases. Table 1 summarizes evidence from nine studies indicating that SGLT2 inhibitors are at least as effective as other diabetic treatments in managing cognitive decline. A randomized controlled clinical trial showed that SGLT2 inhibitors did not induce reduction of cognitive performance in T2DM patients [115]. Four studies reported a reduced risk of cognitive impairment in diabetic patients following treatment with these inhibitors [116–119]. Mone et al. performed a prospective trial and observed cognitive benefits from empagliflozin in frail old patients with diabetes after one month of treatment. They found significant beneficial effects of empagliflozin on cognitive and physical impairment in frail older adults [120]. Another prospective trial demonstrated effective improvement in language domains and executive functioning with treatment with SGLT2 inhibitors for more than 3 years through more than 6 years of follow-up [121]. Additionally, a randomized controlled trial and a single-arm study have corroborated the positive impact of SGLT2 inhibitors on the cognitive function of elderly patients with T2DM and non-diabetic individuals aged 55 or older, respectively [122, 123]. These findings suggest the potential of SGLT2 inhibitors to treat cognitive disorders. Nonetheless, potential side effects, such as urinary tract infections and electrolyte imbalances, warrant consideration (Fig. 4). In addition, in real-world diabetes management, various agents are often used in combination with SGLT2 inhibitors, leading to a more complex interpretation of the observations. A limitation of observational clinical trials is that it is difficult to analyze individual drug effects or make comparisons between them [124].

**Table 1:** Clinical trials of SGLT2 inhibitors

Brief cohort description	Patients	Results	Side effects/study limitations	References
Participants received a maximum dose of metformin as a stable regime for three months, along with addition of SGLT2 inhibitors (canagliflozin 300 mg/day, empagliflozin 25 mg/day, and dapagliflozin 10 mg/day) or incretins (liraglutide at doses of up to 1.8 mg/day, vildagliptin at 100 mg/day, sitagliptin 100 mg/day, and linagliptin 5 mg/day)	Older patients with type 2 diabetes (*n* = 39)	SGLT2 inhibitors are not inferior to enteral insulin therapy in preventing cognitive decline in elderly and diabetic patients. Cognitive condition did not change significantly in either group during the 12-month treatment period	Increased risk of genitourinary infections due to glycosuria	[115]
DPP4 inhibitors and SGLT2 inhibitors started between July 1, 2016, and March 31, 2021	Diabetic individuals (*n* = 106,903)	Over a mean follow-up of 2.80 years following cohort entrance, SGLT2 inhibitors were associated with a decreased incidence of dementia (14.2/1000 person-years; aHR 0.80 [95% CI 0.71–0.89]) compared to DPP-4 inhibitors	Short average follow-up time	[116]
Treatment with SGLT2 inhibitors and DPP4 inhibitors	Type 2 diabetic patients in Hong Kong, China (*n* = 51,460)	SGLT2 inhibitors dramatically lowered the risk of dementia, Parkinson's disease, and cerebrovascular mortality compared to DPP4 inhibitors	Patient medication adherence was uncertain and the trial lacked consideration of lifestyle-induced cardiovascular risk factors	[117]
Standard treatment with and without addition of 10 mg of empagliflozin, 6 months of follow-up	119 patients with diabetes and CKD (*n *= 59 patients receiving empagliflozin plus standard therapy, *n *= 60 with no empagliflozin)	Empagliflozin treatment significantly ameliorated cognitive impairment	Small sample size	[118]
Empagliflozin treatment, three-month follow-up	Consecutive hypertensive and diabetic elderly frail patients (*n* = 150)	Empagliflozin significantly improved the MoCA score and the 5-m Gait Speed in patients	Relatively short follow-up and enrollment of only Caucasian patients	[119]
Treatment with empagliflozin, metformin, or insulin, with follow-up during March 2021 to October 2021	Old adults with HFpEF and diabetes (*n *= 162)	Empagliflozin significantly improved both physical and cognitive impairment	Short follow-up time and relatively small study population	[120]
A prospective cohort study with a follow-up up to 6.4 years, with 138 patients treated with SGLT2 inhibitors	476 T2DM patients aged 60.6 ± 7.4 years	SGLT2 inhibitor use for ≥ 3 years was positively associated with RBANS score increase globally and in language domain	Small sample size	[121]
Dapagliflozin in addition to instruction in cognitive behavior. The starting dosage was one morning dose of 5 mg/time. If the patient required blood glucose management and was tolerant to dapagliflozin, the dose was increased to 10 mg/time, once a day	Senior T2DM patients with mild cognitive impairment (*n* = 96)	Dapagliflozin combined with cognitive behavioral training significantly improved cognitive function, diabetes management self-efficacy, and quality of life in elderly patients with T2DM and mild cognitive impairment. The scores of C-DMSES, ADL, and MMSE of both groups were higher than those before the intervention, and compared with the control group, the scores of the experimental group were significantly higher. The QOL-AD scores of both groups gradually increased, and compared with the control group, the QOL-AD scores of the experimental group were significantly higher at 3 and 6 months after intervention	Long-term safety of dapagliflozin was not addressed in the study and the cognitive-behavioral training approach in the study was relatively homogenous	[122]
A single-arm clinical trial with 14 days of oral empagliflozin (25 mg every morning before breakfast)	Non-diabetics who were 55 years or older (*n* = 21)	Empagliflozin increased the levels of fatty acids and β-hydroxybutyric acid while lowering brain glutamine and glutamate concentrations, which is beneficial in the context of Alzheimer's disease and related dementias	Small sample size	[123]

*SGLT2* sodium-glucose cotransporter-2, *DPP4* dipeptidyl peptidase-4, *HFpEF* heart failure with preserved ejection fraction, *T2DM* diabetes mellitus type 2, *C-DMSES* the Chinese version of the diabetes management self-efficacy, *ADL* activity of daily living, *MMSE* mini-mental state examination, *QOL-AD* quality of life in Alzheimer’s disease, *MoCA* Montreal cognitive assessment

## Challenges of using SGLT2 inhibitors for treatment of cognitive disorders

SGLT2 inhibitors exhibit potential neuroprotective effects across various neurodegenerative disorders, including AD and Parkinson's disease [17, 84]. However, several important questions remain unanswered. First, SGLT2 inhibitors have been reported with side effects including genitourinary infections, ketoacidosis, and fractures [125], and the consequences of their extended use have not been thoroughly studied. Recent evidence suggests an association of SGLT2 inhibitors with acute kidney injury and an elevated risk of bladder cancer development, which highlights the incomplete understanding of their long-term safety profile for cognitive impairment therapy [126]. Second, clinical research evaluating the therapeutic efficacy of SGLT2 inhibitors on cognitive impairment is limited. Most studies were conducted on animal models. The short lifespan of such models prevents adequate evaluation of the efficacy of SGLT2 inhibitors for cognitive impairment. To date, only a handful of clinical trials have investigated the long-term effects of SGLT2 inhibitor usage, leaving their sustained therapeutic value and efficacy in prolonged treatments unresolved. Third, while existing research concentrates on the efficacy of SGLT2 inhibitors for cognitive impairment in diabetic patients, there is a pressing need for studies on the variations in treatment outcomes for cognitive impairments stemming from diverse etiologies [127]. A small number of ongoing clinical trials are addressing the effects of SGLT2 inhibitors on cognitive impairments due to non-diabetic causes, including stroke (ClinicalTrials.gov identifier NCT05565976) and other non-diabetic conditions such as Alzheimer's patients without diabetes (NCT05081219). Fourth, although recent research indicates that SGLT2 inhibitors may prevent the progression of cognitive impairment [115, 128], studies on their ability to reverse established damage are limited. Wang et al. demonstrated that SGLT2 inhibitor-induced normalization of hyperglycemia can reverse cerebrovascular dysfunction and cognitive deficits in chronically hyperglycemic rats; however, evidence in human patients is lacking [6]. Finally, the mechanisms underlying the effects of SGLT2 inhibitors have not been completely understood. Regarding the known side effects of SGLT2 inhibitors, including fractures and ketoacidosis, further research into their specific mechanisms may yield strategies to mitigate these adverse effects through drug combinations. For instance, fractures could be associated with elevated phosphate, fibroblast growth factor 23, and parathyroid hormone levels, along with reduced 1,25-dihydroxy vitamin D concentrations or a decline in blood volume attributable to SGLT2 inhibitors [129]. Derived from these mechanisms, one can hypothesize that combining SGLT2 inhibitors with oestriol or adjusting their dosage could mitigate their side effects.

## Prospects of using SGLT2 inhibitors for treatment of cognitive disorder

SGLT2 inhibitors, primarily prescribed for diabetes management, have recently expanded clinical use to cardiorenal diseases [130]. Concurrently, emerging research has highlighted their potential in treating cognitive disorders. The diverse therapeutic applications and multiple action pathways of SGLT2 inhibitors indicate their promising scalability in clinical settings, with ongoing trials substantiating their viability for cognitive disorder interventions [120, 122]. As polypharmacy is common in chronic disease management, exploring the interactive effects of SGLT2 inhibitors with other medications is of significant interest and warrants further exploration. The synergistic potential based on the pharmacodynamics of SGLT2 inhibitors and the pathophysiology of cognitive disorders is illustrated in Table 2.

**Table 2:** Potential of combining SGLT2 inhibitors with other drugs to improve cognitive impairment

Types of drugs used in combination	Name	Effect	References
Insulin sensitizer	Metformin	Improves insulin resistance and exerts potential neuroprotective, neurotrophic, and neurogenic stimulatory effects that synergize with SGLT2 inhibitors to improve cognitive impairment	[131]
	Thiazolidinediones	Increases insulin receptors to improve insulin sensitivity and also works with SGLT2 inhibitors to improve lipid metabolism disorders and decrease brain inflammation	[132]
Antihyperlipidemic drug	Statins	Prevents vascular sclerosis in the brain by lowering blood lipids, stabilizing and reducing atherosclerotic plaques, as well as reducing the inflammatory response. Synergizes with SGLT2 inhibitors to improve cerebral hemodynamics	[133]
	Fibrates	Reduces total plasma cholesterol and triglycerides, attenuates increased amyloid production due to disorders of lipid metabolism, has anti-inflammatory effects, and improves cerebral hemodynamics in combination with SGLT2 inhibitors	[134, 135]
Angiotensin-converting enzyme inhibitor	Captopril tablets	Increases local bradykinin concentrations, which in turn increases nitric oxide and prostacyclin production, leads to vasodilatation to improve local blood flow and synergistically protects the cerebral vasculature with SGLT2 inhibitors	[136]
Neurotrophin	Acetylcarnitine	Promotes the production of neurotrophic factors and synaptic growth to facilitate nerve regeneration, producing synergistic neuroprotective effects with SGLT2 inhibitors	[137]
Antibacterial drug	Acetaminophen	Reduces neuroinflammation in AD and exerts synergistic neuroprotection with SGLT2 inhibitors	[138]
Antioxidant	Vitamin E	Ameliorates oxidative stress by trapping free radicals, among other things, which improves AD and produces synergistic antioxidant protection with SGLT2 inhibitors	[139]
	Vitamin C	When used with SGLT2 inhibitors, it reduces amyloid plaque formation and can quench reactive oxygen species, which protects mitochondria and reduces the level of oxidative stress in the brain	[140]
	Probucol	Has total cholesterol-lowering and antioxidant effects and may synergize with SGLT2 inhibitors to protect cerebral blood vessels	[141]
GLP-1 agonist	Liraglutide	Prevents memory impairment and synaptic loss, reduces β-amyloid plaque load and microglia-induced inflammation, and enhances synaptic neuroplasticity. May synergise with SGLT2 inhibitors to protect neurons	[142]

*SGLT2* sodium-glucose cotransporter-2, *AD* Alzheimer’s disease, *GLP-1* glucagon-like peptide-1

First, the combined use of SGLT2 inhibitors with insulin sensitizers is advantageous. Brain insulin resistance is a pivotal factor in AD pathogenesis [143], and SGLT2 inhibitors have been shown to enhance cerebral insulin sensitivity by mitigating brain inflammation, apoptosis, and oxidative stress, thereby ameliorating AD progression. Combining these inhibitors with insulin sensitizers could potentially amplify this benefit. Notably, evidence suggests that a regimen combining dapagliflozin and velagliflozin effectively prevents cognitive decline in cases of obesity-related insulin resistance [24]. Second, SGLT2 inhibitors can be combined with lipid-lowering drugs. Diabetes accelerates the aging of blood vessels in the brain, which leads to impaired cognitive function [58, 59]. Statins, on the other hand, can prevent vascular sclerosis by lowering cholesterol levels and inhibiting other downstream products of the mevalonate pathway, so they can synergize with SGLT2 inhibitors to improve cerebral hemodynamics and thus improve cognitive impairment [133]. Additionally, pairing SGLT2 inhibitors with angiotensin-converting enzyme (ACE) inhibitors presents a therapeutic strategy. ACE inhibitors facilitate vasodilation and enhance cerebral blood flow by elevating local bradykinin levels, leading to increased production of nitric oxide and prostacyclin [136]. Such a combination may offer a synergistic effect on cerebral vascular protection. Furthermore, oxidative stress, which contributes to AD through mechanisms like Aβ deposition, tau hyperphosphorylation, and neuronal damage, is a critical target [137]. Antioxidants, by neutralizing free radicals, can mitigate oxidative stress, potentially reducing AD pathology. Vitamin C, a potent antioxidant, may treat cognitive impairment by suppressing the expression of pro-inflammatory genes, diminishing neuroinflammation, and preventing Aβ fibril formation [144], enhancing the therapeutic effect when used with SGLT2 inhibitors. SGLT2 inhibitors can also be used in combination with glucagon-like peptide-1 (GLP-1) agonists. As a diabetic therapeutic agent, GLP-1 agonists have also shown neurotrophic and neuroprotective effects. GLP-1 receptors are present on neuronal soma and dendrites and are significantly expressed in the hippocampus, hypothalamus, cerebral cortex, and olfactory bulb [145]. GLP-1 agonists are capable of rescuing cognitive dysfunction by reducing plaque load, synaptic loss, and neuronal inflammation [146, 147], and thus may exert neuroprotective effects in synergy with SGLT2 inhibitors. Additionally, SGLT2 inhibitors may also be integrated with neurotrophic substances, anti-inflammatory agents, and various other drugs to establish a multifaceted approach to neuroprotection in cognitive disorders.

Nevertheless, these prospects of combinational applications are raised based on the pathways of action of SGLT2 inhibitors on cognitive impairment. More studies are needed to evaluate their safety and therapeutic efficacy.

## Conclusion

The multifaceted effects of SGLT2 inhibitors render them a promising therapeutic candidate for cognitive disorders. In this review, we discuss the roles of SGLT2 in neural function and the diabetes-cognition nexus. Clinical trials have shown cognition-improving effects of SGLT2 inhibitors. The mechanisms underlying the effects include reduction of oxidative stress and neuroinflammation, attenuation of amyloid burdens, enhancement of neuronal plasticity, and improved cerebral glucose utilization. More clinical trials are needed to fully understand the long-term therapeutic safety and effectiveness of SGLT2 inhibitors. The effects of SGLT2 inhibitors on cognitive impairments due to non-diabetic causes also need to be explored. Based on the pharmacodynamics of SGLT2 inhibitors and the pathophysiology of cognitive diseases, this review also demonstrated the synergistic potential of SGLT2 inhibitors and suggested future research avenues.

## Data Availability

Not applicable.

## Abbreviations

SGLT2: Sodium-glucose cotransporter-2
BBB: Blood–brain barrier
AD: Alzheimer’s disease
NLRP3: NOD-like receptor thermal protein domain associated protein 3
BDNF: Brain-derived neurotrophic factor
NGF: Nerve growth factor
GDNF: Glial cell line-derived neurotrophic factor
Aβ: Amyloid β
AMPK: Adenosine monophosphate-activated protein kinase
APP: Amyloid precursor protein
SOD: Superoxide dismutase
GSH: Glutathione
ROS: Reactive oxygen species
TG: Triglycerides
NF-κB: Nuclear factor κ-light chain enhancer of activated B cells
STAT: Signal transducer and activator of transcription
TNF-α: Tumor necrosis factor-α
T2DM: Type 2 diabetes mellitus
PI3K: Phosphatidylinositol 3-kinase
ACE: Angiotensin-converting enzyme
RAGE: Receptor for advanced glycosylation end products
NOX2: NADPH oxidase 2
mTOR: Mammalian target of rapamycin
GLP-1: Glucagon-like peptide-1
